# Practical Model for Residual/Recurrent Cervical Intraepithelial Lesions in Patients with Negative Margins after Cold-Knife Conization

**DOI:** 10.3390/jcm11195634

**Published:** 2022-09-24

**Authors:** Wei Chen, Yajie Dong, Lu Liu, Lin Jia, Lihua Meng, Hongli Liu, Lili Wang, Ying Xu, Youzhong Zhang, Xu Qiao

**Affiliations:** 1 School and Hospital of Stomatology, Cheeloo College of Medicine, Shandong University & Shandong Key Laboratory of Oral Tissue Regeneration & Shandong Engineering Laboratory for Dental Materials and Oral Tissue Regeneration, Jinan 250012, China; 2 Department of Obstetrics and Gynecology, Qilu Hospital, Cheeloo College of Medicine, Shandong University, Jinan 250012, China; 3 Key Laboratory of Gynecologic Oncology of Shandong Province, Jinan 250012, China; 4 Department of Biomedical Engineering, School of Control Science and Engineering, Shandong University, Jinan 250061, China

**Keywords:** cold-knife conization, residual/recurrent, practical model, machine learning

## Abstract

Objective: This study aimed to identify reliable risk factors for residual/recurrent cervical intraepithelial lesions in patients with negative margins after cold-knife conization. Methods: A total of 2352 women with HSILs (high-grade squamous intraepithelial lesions) with negative margins who underwent cold-knife conization between January 2014 and December 2020 were included; in total, 1411 women were assigned to the development cohort, and 941 women were assigned to the validation cohort. Multivariate logistic regression was used to build four predictive models based on the different combinations of follow-up data (Model A: preoperative factors; Model B: first-follow-up data; Model C: second-follow-up data; Model D: data from both follow-ups). The accuracy, sensitivity, specificity, false-positive rate (FPR), false-negative rate (FNR), and area under the receiver operating characteristic curve (AUC) were evaluated on the validation cohort. The predictive power of risk factors was further validated using six machine learning algorithms. Results: Model D demonstrated the highest AUC of 0.91 (95% CI, 0.87 to 0.96) in the validation cohort, whereas Models A, B, and C achieved AUCs of 0.69 (95% CI, 0.59 to 0.78), 0.88 (95% CI, 0.80 to 0.95), and 0.89 (95% CI, 0.81 to 0.97) respectively. The six machine learning methods achieved consistent results. Kaplan-Meier (KM) survival curves demonstrated that our models could effectively stratify patients with all models (*p* < 0.05 for all models). Conclusion: Our model, which is based on preoperative and follow-up factors, can serve as a complementary screening procedure for the early detection or prediction of recurrence after cold-knife conization in HSIL patients.

## 1. Introduction

Human papillomavirus (HPV) infection is a high-risk factor for cervical cancer. Infected women may develop cervical cancer after several years or even more than ten years [1]. Cervical intraepithelial neoplasia (CIN) is a precancerous lesion of cervical cancer and can be divided into low-grade squamous intraepithelial lesions (LSILs) and high-grade squamous intraepithelial lesions (HSILs). An estimated 30% of HSIL cases may progress to cervical cancer [2], and cervical cancer can be prevented by screening and treating HSILs. Excisional therapy is commonly used to treat HSIL and includes loop electrosurgical excision procedures (LEEPs or LLETZs), cold-knife conization, and laser cone biopsy [3]. The failure rate of excisional treatment, defined as persistent or recurrent HSILs or worsening disease, is reported to be between 4% and 18% [4], and the majority of these cases occur within two years of primary treatment [5]. The risk of developing cervical cancer after cervical conization in HSIL patients is five times that of the general population [6]. Additionally, women treated for HSILs may have an increased risk of recurrent CIN and cervical cancer for up to 25 years [7,8].

Therefore, we need standardized management strategies for patients after surgery. Postoperative follow-up should occur in women with HSIL according to the conization margin status. Patients with negative margins typically undergo cytology or HPV testing after 6, 12, and 24 months. If the test results are abnormal, they are referred for colposcopy. Women with positive margins typically undergo colposcopy-guided biopsy and cytology testing within 4–6 months [9,10]. The problem of treatment failure in HSILs has been studied for decades, but uncertainty and debate remain as to which factor or combination of factors is the most accurate predictor of treatment failure. Several factors are considered to be a risk for treatment failure after excisional treatment, including age, smoking, size, the severity of lesions, high-risk human papillomavirus (HR-HPV) type, and HR-HPV persistence [11,12].

Regardless of the strategy used, follow-up outcomes are suboptimal due to poor patient compliance. A study in Australia found that only 26.6% of women completed a combined cytology and HPV test within 12 months of CIN resection treatment [10]. Overall, the assessment of postoperative recurrence in each patient is very difficult. In addition, few studies have examined patients with negative surgical margins, and drawing valuable conclusions is consequently challenging. This study retrospectively assessed risk factors for residual/recurrent cervical intraepithelial lesions in women with negative margins after cold-knife conization and established a predictive model. We aimed to identify reliable risk factors for residual/recurrent cervical intraepithelial lesions in patients with negative margins after cold-knife conization, thereby enabling risk management for patients, reducing patient anxiety to a certain extent, and promoting timely treatment.

## 2. Materials and Methods

### 2.1. Study Design

**Figure 1:**
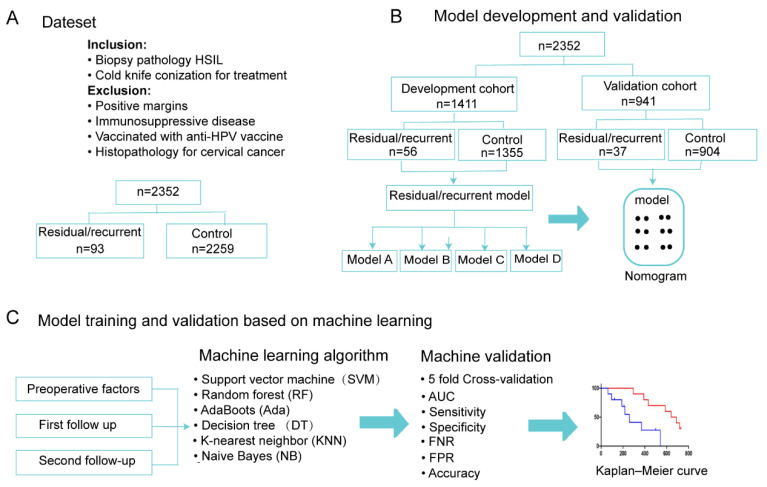
Flowchart of the study. (**A**) Overall dataset; (**B**) model development and validation; (**C**) model training and validation based on six machine learning methods. HSIL, high-grade squamous intraepithelial lesion; AUC, area under the curve; FNR, false-negative rate; FPR, false-positive rate; SVM, support vector machine; RF, random forest; Ada, AdaBoost; DT, decision tree; KNN, k-nearest neighbor; NB, naive Bayes.

A summary of this retrospective study is shown in Figure 1. We searched for women with a histological diagnosis of HSILs upon colposcopy biopsy who underwent cold-knife conization or LEEP at Shandong University Qilu Hospital. The study obtained approval from the Ethics Committee of Qilu Hospital of Shandong University (KYLL-202107-134) and obtained a waiver for informed consent. Our study was a retrospective study; we included patients who underwent cervical conization between January 2014 and December 2020, of whom 2352 patients underwent CKC surgery and 291 patients underwent LEEP surgery. Patients were followed up until December 2021, with a maximum follow-up period of 7 years. The exclusion criteria were as follows: diagnosis of other histological types; patient received HPV vaccination; no follow-up data; positive margins after cold-knife conization; and immunosuppression.

### 2.2. Follow-Up

Patients had their first follow-up 4–6 months after cold knife conization and their second follow-up 10–12 months after surgery. Liquid-based cytology and HPV testing were performed at each follow-up. A cervical biopsy was performed if women had abnormal cytology results (e.g., atypical squamous cells of indeterminate significance or more severe lesions), positive HPV results, or abnormal results from colposcopy. Specimens were collected for HPV testing with a Digene kit (Digene, Gaithersburg, MD, USA) or Roche Cobas 4800 kit (Roche Molecular, Branchburg, NJ, USA). Digene produces quantitative results for 13 HR-HPV genotypes (HPV 16, 18, 31, 33, 35, 45, 51, 52, 56, 58, 59, and 68). A relative light unit/cutoff (RLU/CO) ratio ≥1.0 indicated a positive result. The qualitative results of Cobas 4800 detect HPV16, HPV18, and 12 other HR-HPV genotypes (HPV 31, 33, 35, 45, 51, 52, 56, 58, 59, 66, and 68).

### 2.3. Criteria for Residual/Recurrent Disease

The criteria for developing residual/recurrent disease were histological examination based on colposcopy biopsy. Histological evidence of CIN (LSIL or HSIL) was considered a residual/recurrent disease. Residual lesions were defined as those diagnosed within the first year of cold-knife conization or LEEP. Cold-knife conization and LEEP were performed in the operating room by experienced gynecologic oncologists. The cervix was exposed and smeared with iodine solution. The cervix was excised in a conical shape 0.5 cm outside the iodine-unstained area, and the height of the cone could reach 2–2.5 cm. Cervical lesions detected after one year were considered recurrences. In this study, we analyze residual disease and recurrence together.

### 2.4. Predictors and Endpoints

The following clinical characteristics were included: age; pregnancy; parity; types of cervical transformation zones; preconization cytology; preconization HPV; endocervical curettage (ECC); first follow-up HPV (F-HPV) after conization; first follow-up cytology (F-TCT) after conization; second follow-up HPV (S-HPV) after conization; second follow-up cytology (S-TCT) after conization; improved (conization histopathology lower than colposcopy biopsy), severe (conization histopathology higher than colposcopy biopsy), and residual disease/recurrence information. Residual disease/recurrence time was described as the time interval from surgery to the first appearance of CIN. Residual disease/recurrence was determined via colposcopy biopsy histology.

### 2.5. Model Construction and Validation

We randomly split all patients into development and validation cohorts in a 6:4 ratio. For the development cohort, we first performed a univariate analysis to screen out statistically significant features; we then constructed logistic regression models to explore the role of clinical factors in prognosis. To demonstrate the importance of follow-up data, we constructed four models, namely, a model based on preoperative factors (Model A), a model based on first-follow-up data and reoperative factors (Model B), a model based on second-follow-up data and reoperative factors (Model C), and a model based on data from these two follow-ups and reoperative factors (Model D).

Each model was built based on stratified 5-fold cross-validation to guarantee generalization ability. At each iteration, four folds of the development cohort were used for model training, and the remaining one fold was used for validation. The role of cross-validation was to select the optimal hyperparameters by maximizing the performance on the validation folds. After the cross-validation procedure, all models were retrained with the entire development cohort and evaluated in the independent validation cohort.

The discrimination power, defined as the agreement between the predicted and actual residual/recurrent disease probability, was used to evaluate the performance of our models. In this study, the discrimination power was estimated using metrics such as accuracy, sensitivity, specificity, false-positive rate (FPR), false-negative rate (FNR), and area under a curve (AUC). In addition, we drew a nomogram, a reliable tool for graphically representing residual/recurrent disease probability. We then used calibration curves to intuitively assess the agreement between the actual residual/recurrent disease and the predicted residual/recurrent disease. Finally, we used decision curves to determine the clinical usefulness of our models.

In recent years, an increasing number of studies have used a variety of machine learning (ML) methods to construct clinical predictive models [13]. In some cases, ML methods can perform better than traditional regression methods due to the complexity of implicit patterns in the data. Therefore, we further used six ML algorithms to validate our selected high-risk factors, including support vector machine (SVM) [14], random forest (RF) [15], AdaBoost (Ada) [16], decision tree (DT) [17], k-nearest neighbor (KNN) [18], and naive Bayes (NB) [19]. These methods are popular machine learning algorithms and have been widely used in clinical prediction models [20,21,22].

Given the increasing number of younger patients choosing LEEP over CKC due to their reproductive needs, we further developed a predictive model for patients who underwent LEEP for HSILs to validate the generalizability of our proposed method. A total of 291 women with HSILs treated with LEEP were included, including 205 patients without residual disease/recurrence and 86 patients with residual disease/recurrence. The model was also validated using various ML algorithms.

### 2.6. Statistical Analysis

The statistical analysis was conducted with R software (version 4.1.0) and Python (version 3.8.8) ML library Scikit-Learn (version 0.24.1) [23]. The distributions of the potential predictive factors were compared between residual disease/recurrence patients and controls using the chi-squared test. The DeLong test was used to assess the differences between ROCs. All tests were two-tailed, and statistical significance was defined as *p* < 0.05.

## 3. Results

### 3.1. Characteristics of Patients Who Underwent Cold-Knife Conization

We included 2352 women who underwent cold-knife conization, including 2259 controls and 93 residual disease/recurrent patients. The median follow-up of patients was 30 months (range: 4–257 months), with 75% of patients being followed for more than 149 months. The median time to patient residual/recurrent disease was 11 months (range: 4–56 months). Patients had their first follow-up 4–6 months after cold-knife conization and a second follow-up 10–12 months after surgery. Therefore, we defined HPV16, HPV18, or HR-HPV RLU/CO > 1000 as positive before treatment. Unavailable TCT and HPV results were defined as unknown. The patient characteristics are summarized in Table 1. According to the chi-squared test results in the development cohort, factors with *p* < 0.05 were included in the modeling analysis.

Due to the different follow-up rates of patients after surgical treatment, we established four predictive models based on preoperative and postoperative follow-up data. Four preoperative risk factors were included in Model A, including pregnancy, ECC, improvement, and preoperative HPV results. At the first follow-up after cold-knife conization, 1311 (55.7%) hrHPV test results and 1080 (45.9%) cytology results were available. At the second follow-up after cold-knife conization, 854 (36.3%) hrHPV test results and 771 (32.8%) cytology test results were available. We combined the first- and second-follow-up data (including HPV and cytology) with preoperative risk factors and established two predictive models (Model B and Model C). Finally, we integrated the first- and second-follow-up data for analysis. At least one positive HPV test and at least one positive cytology test were used as postoperative risk factors and were combined with preoperative risk factors to construct Model D.

### 3.2. Model Development and Validation

**Figure 2:**
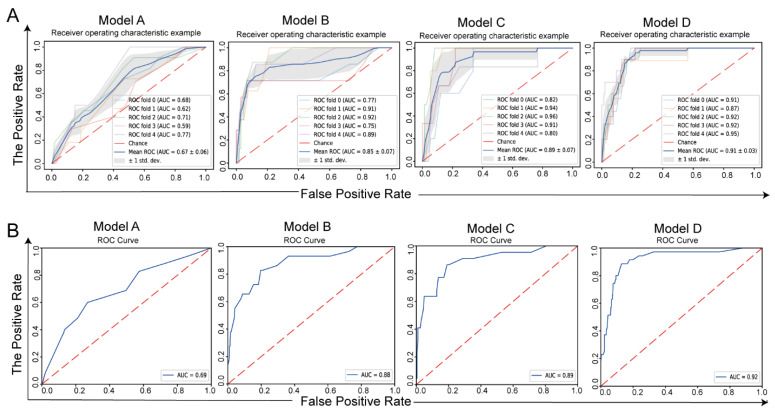
ROC curves of the four models. (**A**) ROC curves of 5-fold cross validation of the development cohort; (**B**) ROC curves of the validation cohort.

The ROC curves of the five-fold cross-validation of the development cohort and the ROC curves of the validation cohort are shown in Figure 2. The mean cross-validation AUC of Model A was 0.67. The mean cross-validation AUCs of Models B and C were 0.85 and 0.89, respectively. Model D had the highest mean cross-validation AUC, 0.91 (Figure 2A). In the validation group, the AUC of Model A was 0.69. The AUCs of Models B and C were 0.88 and 0.89, respectively. Consistent with the results of the development group, Model D had the highest AUC, at 92% (Figure 2B). Therefore, Model D had the best discriminative power. In addition, other metrics, such as accuracy, sensitivity, specificity, FPR, and FNR, are listed in Table 2. The five-fold cross-validation in the development cohort predicted residual disease/recurrence with an accuracy of 0.58 ± 0.13 (AUC = 0.58 ± 0.13) for Model A, 0.86 ± 0.08 (AUC = 0.85 ± 0.07) for Model B, 0.81 ± 0.08 (0.89 ± 0.07) for Model C, and 0.85 ± 0.05 (AUC = 0.92 ± 0.01) for Model D. In the validation cohort, the accuracy of Model A was 0.72 (AUC = 0.69); the accuracy of Model B was 0.81 (AUC = 0.88); the accuracy of Model C was 0.82 (AUC = 0.89); and the accuracy of Model D was 0.78 (AUC = 0.91).

**Figure 3:**
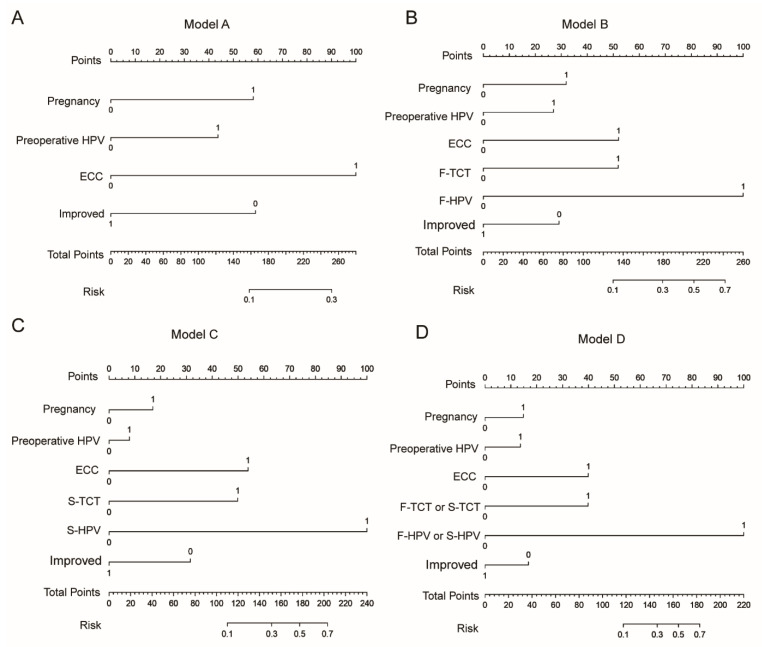
The nomograms of four models: (**A**) Model A; (**B**) Model B; (**C**) Model C; (**D**) Model D.

**Figure 4:**
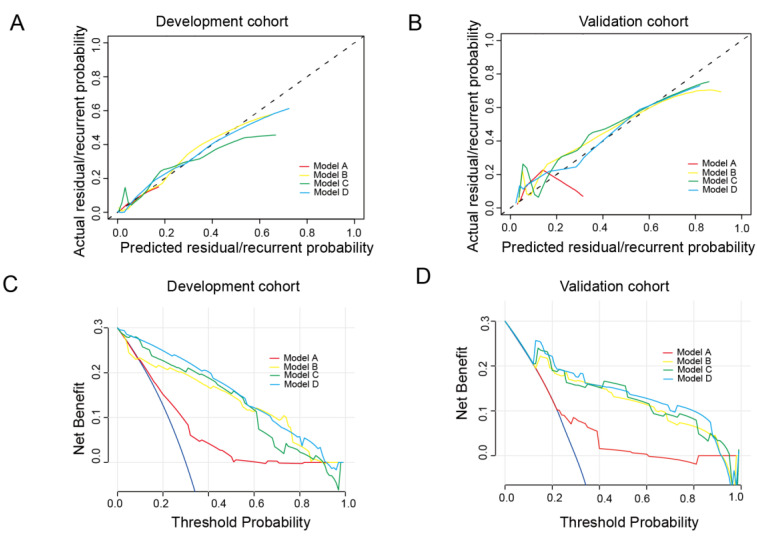
The calibration curves for the development cohort (**A**) and the validation cohort (**B**). The decision curves for the development cohort (**C**) and the validation cohort (**D**).

Four nomograms built based on the regression coefficients of the models are shown in Figure 3. The calibration curves are shown in Figure 4A. The calibration curve demonstrated satisfactory consistency between the predicted residual/recurrent disease and the actual observed residual/recurrent disease in both the development and validation cohorts, especially for Model D. The decision curves of the nomograms are presented in Figure 4B. Model D provided a higher net benefit than the other models in both the development and validation cohorts.

### 3.3. Model Training and Validation Based on ML Methods

**Figure 5:**
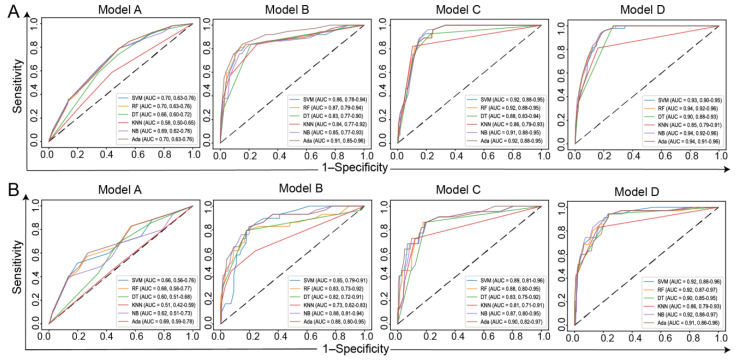
The ROCs of six ML methods. (**A**) ROCs of the development cohort; (**B**) ROCs of the validation cohort.

We further validated our risk factors using six ML methods, and the performance metrics of the validation cohort are listed in Table 3. For Model A, significant differences were observed between the AUC of LR and those of DT, KNN, and NB (*p* < 0.05), but not significantly different when compared with other ML methods. The mean AUC of all methods was 0.63. For Model B, significant differences were observed between the AUCs of LR and KNN (*p* < 0.05), but they were not significantly different when compared with other ML methods. The mean AUC of all methods was 0.84. For Model C, significant differences were observed between the AUC of LR and those of DT and KNN (*p* < 0.05), but they were not significantly different when compared with other ML methods. The mean AUC of all methods was 0.87. For Model D, significant differences were observed between the AUCs of LR and KNN (*p* < 0.05), but they were not significantly different when compared with other ML methods. The mean AUC of all methods was 0.90. The ROCs of all ML methods for the development and validation cohorts are shown in Figure 5.

### 3.4. Kaplan–Meier Estimates

**Figure 6:**
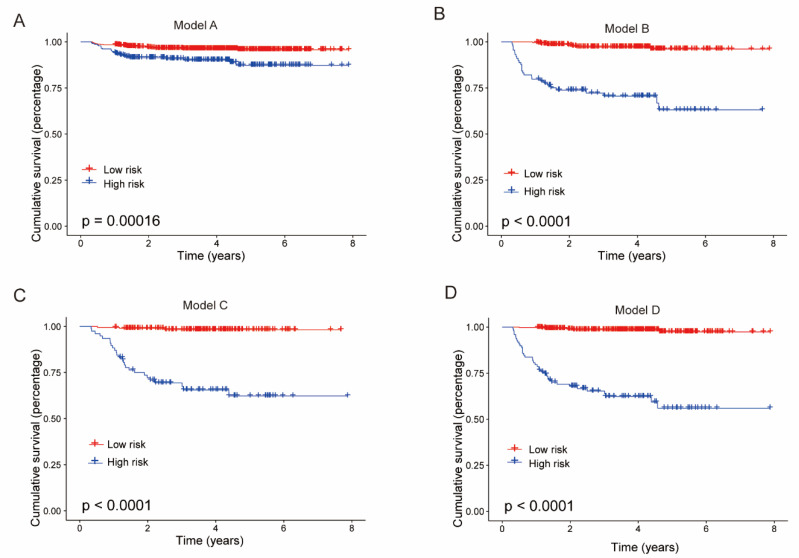
KM survival curves of different models in the validation cohort. (**A**) Model A; (**B**) Model B; (**C**) Model C; (**D**) Model D.

To further demonstrate the effectiveness of our model for patient stratification, we divided patients into high- and low-risk groups based on predicted outcomes and plotted Kaplan–Meier (KM) survival curves. A log-rank test was used to assess the statistical significance of the differences between the high- and low-risk groups. As shown in Figure 6, all models were able to effectively stratify patients, and Model D had the best performance (*p* < 0.0001).

### 3.5. Model for LEEP

A total of 291 women with HSILs treated with LEEP were included, including 205 patients without residual disease/recurrence and 86 patients with residual disease/recurrence. All enrolled patients had a follow-up time of more than 2 years, and the clinical characteristics are shown in Table S1. We randomly split all patients into development and validation cohorts in a 6:4 ratio and analyzed these data using methods consistent with the analysis of CKC patients. Preoperative HPV status, transformation-zone type, HPV status within one year after surgery, and TCT status within one year after surgery were identified as risk factors. Figure S1 shows the ROC curves achieved using the traditional LR method and the six machine learning methods for the development and validation cohorts. The predictive performance of different methods in the validation cohort are listed in Table S2. All methods achieved consistent results, with AUCs ranging from 0.84 to 0.91. The nomograms built based on the regression coefficients of the model are shown in Figure S2. The calibration curves and decision curves are shown in Figure S3. It can be seen that both the LEEP-based model and the CKC-based model had excellent predictive performance.

## 4. Discussion

This study compared potential residual/recurrent disease predictors between patients with residual/recurrent HSILs and controls who displayed different characteristics. Furthermore, we developed a practical identification model based on eight readily available preoperative and postoperative factors to effectively identify residual/recurrent HSIL patients. Benefiting from the advantages of feasibility and almost zero cost, this model has the potential to compensate for the current deficiencies in cervical cancer screening, especially in underdeveloped areas where screening facilities are lacking. ML technology has emerged as an efficient computer algorithm for identifying patterns in large data sets with many variables and facilitating data-driven predicting or categorical modeling [24]. We validated the model using ML algorithms. Our findings suggest that this excellent prognostic risk assessment model can be used in clinical practice as a potential assessment tool for patients with residual/recurrent HSILs after treatment. We believe that follow-up testing at 6 and 12 months after cold-knife conization could better assess residual/recurrent disease. However, in underdeveloped areas without follow-up conditions, preoperative factors can also be used to predict the treatment effect in HSIL patients.

In our study, 4% of HSIL patients experienced “treatment failure”, consistent with a prevalence of 3.5–12% recently reported by several authors [25,26,27]. Our study found that some pre-follow-up factors were associated with treatment failure, including HPV16, HPV18, or HR-HPV RLU/CO > 1000, more than three pregnancies, positive ECC, and improved lesions. HPV clearance usually occurs within three months from surgery, and HPV16 and 18 did not clear rapidly [28,29]. The HPV genotype is a predictor of relapse, with HPV16 causing more HSIL relapses than other high-risk HPV types [30,31]. The high-risk HPV DNA load is highly correlated with the development of cervical lesions. Higher viral loads increase the likelihood of viral DNA integration into host-cell DNA [32]. The viral load of HR-HPV is associated with extensive cervical lesions that are more likely to recur [29]. Patients with a high pretreatment HR-HPV viral load should be considered at risk of developing residual/recurrent disease and may require more rigorous follow-up. In our study, 68.2% (60/88) of patients who failed HSIL surgery had HPV16, HPV18, or HR-HPV RLU/CO > 1000.

The grade of CIN lesions directly correlated with the risk of developing invasive cervical cancer. However, the relationship between lesion grade and residual/recurrent disease after conization was controversial. In our study, 26.9% (25/93) of patients had elevated pathological grades after conization (*p* = 0.326; OR, 1.3; 95% CI, 0.82–2.08). However, we found that decreased pathological grade after the conization of HSILs was a protective factor for surgical failure (*p* = 0.04; OR, 0.61; 95% CI, 0.39–0.96).

ECC is an effective pathological examination method that has attracted increasing attention in recent years. Cuello et al. [33] believe that ECC has irreplaceable value in the diagnosis of cervical lesions. In addition, a positive ECC result is a predictor of persistent/recurrent disease after LEEP treatment. Our study found that ECC positivity indicated a residual disease/recurrence risk (OR = 4.2). Regarding selection bias in ECC patients, clear expert consensus or guidelines are lacking at home or abroad for reference. Age and transition-zone category were the main factors affecting the detection rate of ECC [3]. In our study, age was not a risk factor for residual disease/recurrence of cervical lesions. However, postmenopausal and type III transformation zones accounted for a higher proportion of residual/recurrent cases (5% vs. 7.5% and 14.5 vs. 19.0%, respectively).

Our study found that having undergone more than three pregnancies was a risk factor for residual/recurrent disease. In previous studies, low pregnancy was found to be a preventive factor for recurrence in patients with positive margins [34]. The high concentrations of estrogen and progesterone during pregnancy can lead to the eversion of the columnar epithelium, which results in HPV infection at the squamocolumnar junction.

Most women treated with hrHPV clear it within six months. These women have a lower risk of developing CIN of grade 2 or higher after treatment than women who have not cleared the virus [29,35]. Studies have shown that the sensitivity of persistent HPV infection at six months after surgery to predict persistent/recurrent lesions is 81–97% [29,36]. Cytology plays an important role in follow-up, and women with three consecutive negative cytology results at 6, 12, and 24 months after treatment have a lower risk of developing CIN3 or higher-risk disease than women with at least one abnormal cytology result [5]. At the 6- and 12-month follow-up after cold-knife conization, any positive HPV test result or cytology test result can be used as a risk factor for predicting residual/recurrent cervical lesions.

Given that more and more young patients choose LEEP instead of CKC due to their reproductive needs, we collected HSIL patients who underwent LEEP and analyzed the prognostic factors affecting recovery. We found that the prognostic factors affecting patients with LEEP surgery were different from those who underwent CKC surgery, mainly in the ECC and transformation zone. This is because of individual differences in patients’ choice of surgical approach. We tend to recommend CKC for more complete resection of the diseased tissue for ECC-positive patients. Therefore, in patients undergoing LEEP surgery, the proportion of patients undergoing ECC is much smaller than that of CKC. In addition, the type III transformation zone is a risk factor affecting patients after LEEP. The transformation zone is the leading site of cervical precancerous lesions. The more visible the parts of the transformation zone are, the greater the chance of exposed lesions and the higher the diagnostic accuracy of biopsy are [37]. The biopsy accuracy of the type III transformation zone is significantly lower than that of type I/II. I In addition, patients with type III transformation zone or ECC-positive patients have a greater chance of residual disease/recurrence after LEEP. Therefore, the type of transformation zone and the extent of the lesions should be considered when choosing surgical methods for HSIL patients to achieve no residual disease.

The strength of our study is the development of four prognostic assessment models for HSIL patients through a unique combination of preoperative and postoperative follow-up factors. For some underdeveloped areas that lack follow-up conditions, preoperative factors can be used to screen patients with residual disease/recurrence tendencies, but the performance of our preoperative prediction model needs to be improved. Our model does not replace co-testing screening and colposcopy after surgery, but our model can advise patients based on their individual differences. According to the model’s prediction results, we recommend the regular follow-up of patients with combined cytology and HPV detection after surgery. In particular, patients with positive results for co-testing screening 6 and 12 months after surgery would benefit from regular screening. Based on the weighted risk factors in the nomogram lists, these models can provide individualized predictions for each patient. Next, we selected the ML algorithm to verify the model’s predictive ability through five-fold cross-validation. For patients with a high-risk factor predicted in the model, we tell the patient to follow up closely and get treated in time. Our study is meaningful in countries without rigorous screening programs. However, our study is subject to some limitations. First, we only collected follow-up data 6 months and 12 months after cold-knife conization; we then expanded the collection of follow-up data according to follow-up rules. Second, our study was a retrospective study, and we only collected less than 50% of the follow-up data, which caused research bias. Third, the predictive models we built lack independent external validation.

## 5. Conclusions

Based on the analysis of preoperative and postoperative factors, this study established four accurate and practical identification models for predicting residual/recurrent disease after cold-knife conization and revealed the risk factors associated with residual/recurrent disease. The identification model can serve as a complementary screening procedure for the early detection or prediction of recurrence after cold-knife conization in HSIL patients, which is especially useful in underdeveloped and remote areas.

## Figures and Tables

**Table 1:** Patients and corresponding clinical features.

	Development Cohort (1411)			Validation Cohort (941)		
Patient Characteristic	No Residual/	Residual/	*p*-Value	No Residual/	Residual/	*p*-Value
	Recurrent CIN (1355)	Recurrent CIN (56)		Recurrent CIN (904)	Recurrent CIN (37)	
Age (years)						
<45	1004	44	0.552	659	26	0.87
≥45	351	12		245	11	
Pregnancy						
<3	904	27	0.007	609	13	<0.001
≥3	451	29		295	24	
Parity						
<2	808	32	0.823	539	19	0.408
≥2	547	24		365	18	
Menopause						
Yes	1288	50	0.109	858	35	1
No	67	6		46	2	
TCT						
<ASCUS	307	11	0.544	226	14	0.222
≥ASCUS	922	43		580	22	
Unknown	126	2		98	1	
HPV16/18 or RLUs > 1000						
Yes	625	38	0.024	341	13	0.477
No	512	15		421	22	
Unknown	218	3		142	2	
Transformation zone						
Type I/II	632	29	0.492	409	22	0.882
Type III	102	7		75	5	
Unknown	621	20		420	10	
ECC						
Positive	36	5	0.02	27	5	0.003
Negative	1319	51		877	32	
Improved						
Yes	565	14	0.019	369	14	0.849
No	790	42		535	23	
Severe						
No	1056	39	0.195	706	29	1
Yes	299	17		198	8	
First follow-up after conization						
HR-HPV negative	585	7	<0.001	382	7	<0.001
HR-HPV positive	166	36		103	25	
Unknown	604	13		419	5	
TCT < ASCUS	611	27	<0.001	378	20	<0.001
TCT >= ASCUS	16	13		6	9	
Unknown	728	16		520	8	
Second follow-up after conization						
HR-HPV negative	384	4	<0.001	242	5	<0.001
HR-HPV positive	114	32		53	20	
Unknown	857	20				
TCT < ASCUS	435	23	<0.001	261	15	<0.001
TCT > ASCUS	13	7		7	10	
Unknown	907	26		636	12	

ASCUS, atypical squamous cells of undetermined significance; CIN, cervical intraepithelial neoplasia; ECC, endocervical curettage; HR-HPV, high-risk human papillomavirus; RLUs, relative light units.

**Table 2:** Diagnostic performance of the development and validation cohorts.

	Threshold	AUC	Sensitivity	Specificity	FPR	FNR	Accuracy
Development-Cohort Cross-Validation							
Model A	0.51 ± 0.12	0.58 ± 0.13	0.73 ± 0.24	0.58 ± 0.15	0.4 ± 0.15	0.2 ± 0.24	0.58 ± 0.13
Model B	0.65 ± 0.15	0.85 ± 0.07	0.88 ± 0.17	0.86 ± 0.09	0.14 ± 0.09	0.1 ± 0.17	0.86 ± 0.08
Model C	0.51 ± 0.24	0.89 ± 0.07	0.97 ± 0.07	0.80 ± 0.09	0.20 ± 0.09	0.0 ± 0.07	0.81 ± 0.08
Model D	0.59 ± 0.15	0.92 ± 0.01	0.94 ± 0.12	0.84 ± 0.06	0.1 ± 0.06	0.0 ± 0.12	0.85 ± 0.05
Validation cohort							
Model A	0.53	0.69 (0.59–0.78)	0.60	0.73	0.27	0.4	0.72
Model B	0.44	0.88 (0.80–0.95)	0.83	0.80	0.20	0.17	0.81
Model C	0.43	0.89 (0.81–0.97)	0.86	0.81	0.19	0.14	0.82
Model D	0.42	0.91 (0.87–0.96)	0.94	0.77	0.23	0.06	0.78

AUC, area under the curve; FPR, false-positive rate; FNR, false-negative rate.

**Table 3:** The predictive performance of different ML methods in the validation cohort.

	AUC	Sensitivity	Specificity	FPR	FNR	Accuracy
**Model A**						
SVM	0.66 (0.56–0.76)	0.51	0.80	0.20	0.49	0.79
RF	0.68 (0.58–0.77)	0.57	0.74	0.26	0.43	0.73
DT	0.60 (0.51–0.68)	0.69	0.52	0.48	0.31	0.53
KNN	0.51 (0.42–0.59)	0.46	0.56	0.44	0.54	0.56
NB	0.62 (0.51–0.73)	0.4	0.86	0.14	0.6	0.84
Ada	0.69 (0.59–0.78)	0.6	0.73	0.27	0.4	0.73
**Model B**						
SVM	0.85 (0.79–0.91)	0.79	0.82	0.18	0.21	0.82
RF	0.83 (0.73–0.92)	0.79	0.82	0.18	0.21	0.82
DT	0.82 (0.72–0.91)	0.79	0.82	0.18	0.21	0.82
KNN	0.73 (0.62–0.83)	0.62	0.76	0.24	0.38	0.75
NB	0.88 (0.81–0.94)	0.83	0.80	0.21	0.17	0.80
Ada	0.88 (0.80–0.95)	0.83	0.80	0.20	0.17	0.81
**Model C**						
SVM	0.89 (0.81–0.96)	0.86	0.80	0.20	0.14	0.81
RF	0.88 (0.80–0.95)	0.86	0.82	0.18	0.14	0.83
DT	0.83 (0.75–0.92)	0.86	0.80	0.20	0.14	0.81
KNN	0.81 (0.71–0.91)	0.72	0.88	0.12	0.27	0.86
NB	0.87 (0.80–0.95)	0.86	0.80	0.2	0.14	0.81
Ada	0.90 (0.82–0.97)	0.86	0.81	0.19	0.14	0.82
**Model D**						
SVM	0.91 (0.87–0.96)	0.94	0.77	0.24	0.06	0.78
RF	0.92 (0.87–0.96)	0.94	0.77	0.23	0.06	0.78
DT	0.90 (0.85–0.95)	0.94	0.76	0.24	0.06	0.78
KNN	0.86 (0.79–0.93)	0.8	0.86	0.14	0.2	0.86
NB	0.92 (0.88–0.97)	0.94	0.77	0.23	0.06	0.78
Ada	0.91 (0.86–0.96)	0.94	0.77	0.23	0.06	0.78

The *p*-value was calculated using the DeLong test by comparing the AUC values between the LR model and other ML models. AUC, area under the curve; SVM, support vector machines; RF random forests; DT, decision tree; KNN, k-nearest neighbor; NB, naive Bayes; Ada, AdaBoost.

## Data Availability

Not applicable.

## Supplementary Materials

The following supporting information can be downloaded at: https://www.mdpi.com/article/10.3390/jcm11195634/s1, Figure S1: (A) ROC curves of the development cohort; (B) ROC curves of the validation cohort; Figure S2: Nomogram list of the proposed model; Figure S3: The calibration curves of the development cohort (A) and the validation cohort (B). The decision curves of the development cohort (C) and the validation cohort (D); Table S1: Patients and corresponding clinical features; Table S2: The predictive performance of different methods in the validation cohort.
