# Supplementary material for: Practical Model for Residual/Recurrent Cervical Intraepithelial Lesions in Patients with Negative Margins after Cold-Knife Conization
Source: J Clin Med. 2022 Sep 24;11(19):5634. doi: 10.3390/jcm11195634 (PMC9573483; doi:10.3390/jcm11195634)
Supplement: Supplementary file 1 [file jcm-11-05634-s001.zip › jcm-1885382-supplementary.pdf]

Table S1. Patients and corresponding clinical features.

| Development cohort (174) | | | | Validation cohort (117) | | |
|---|---|---|---|---|---|---|
| Patient characteristics | No Residua/ Recurrent CIN (122) | Residua/ Recurrent CIN (52) | P value | No Residua/ Recurrent CIN（83） | Residua/Recurrent CIN（34） | P value |
| **Age（years）** | | | | | | |
| <45 | 87 | 36 | 0.925 | 63 | 24 | 0.715 |
| ≥45 | 35 | 16 | | 20 | 10 | |
| **Pregnancy** | | | | | | |
| <3 | 57 | 25 | 1 | 35 | 17 | 0.569 |
| ≥3 | 65 | 27 | | 48 | 17 | |
| **Parity** | | | | | | |
| <2 | 78 | 39 | 0.212 | 64 | 20 | 0.077 |
| ≥2 | 44 | 13 | | 19 | 14 | |
| **Menopause** | | | | | | |
| No | 107 | 43 | 0.524 | 80 | 28 | 0.028 |
| Yes | 15 | 9 | | 3 | 6 | |
| **TCT** | | | | | | |
| <ASCUS | 46 | 16 | 0.483 | 32 | 8 | 0.18 |
| ≥ASCUS | 76 | 36 | | 51 | 26 | |
| **HPV16/18 or RLU>1000** | | | | | | |
| No | 74 | 17 | 0.001 | 47 | 10 | 0.013 |
| Yes | 48 | 35 | | 36 | 24 | |
| **Transformation zone** | | | | | | |
| Type I/II | 10 | 36 | <0.001 | 6 | 27 | <0.001 |
| Type III | 111 | 16 | | 77 | 7 | |
| **ECC** | | | | | | |
| Negative | 117 | 50 | 1 | 81 | 32 | 0.705 |
| Positive | 5 | 2 | | 2 | 2 | |
| **Improved** | | | | | | |
| No | 35 | 23 | 0.070 | 22 | 15 | 0.101 |
| Yes | 87 | 29 | | 61 | 19 | |
| **Severe** | | | | | | |
| No | 29 | 17 | 0.198 | 26 | 10 | 1 |
| Yes | 93 | 39 | | 57 | 24 | |
| **Fllow-up** | | | | | | |
| HR-HPV Negative | 37 | 5 | 0.007 | 24 | 3 | 0.036 |
| HR-HPV Positive | 85 | 46 | | 29 | 31 | |
| TCT <ASCUS | 111 | 35 | <0.001 | 74 | 24 | 0.028 |
| TCT >=ASCUS | 11 | 17 | | 9 | 0 | |

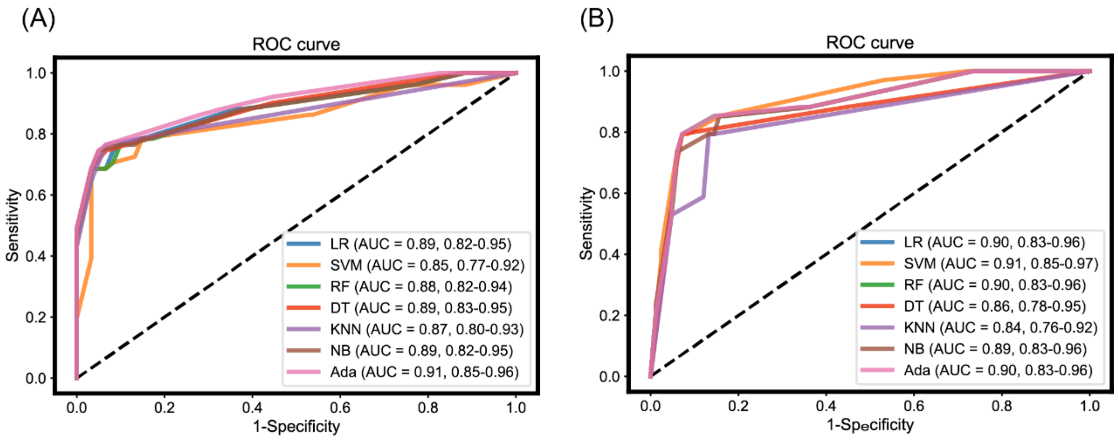


**Figure S1.** (A) ROC curves of the development cohort; (B) ROC curves of the validation cohort.

Table S2. The predictive performance of different methods in the validation cohort.

| | AUC | Sensitivity | Specificity | FPR | FNR | accuracy |
|---|---|---|---|---|---|---|
| **Model A** | | | | | | |
| LR | 0.90 (0.83-0.96) | 0.79 | 0.93 | 0.07 | 0.21 | 0.89 |
| SVM | 0.91 (0.85-0.97) | 0.79 | 0.93 | 0.07 | 0.21 | 0.89 |
| RF | 0.90 (0.58-0.77) | 0.79 | 0.93 | 0.07 | 0.21 | 0.89 |
| DT | 0.86 (0.51-0.68) | 0.79 | 0.93 | 0.07 | 0.21 | 0.89 |
| KNN | 0.84 (0.42-0.59) | 0.79 | 0.87 | 0.13 | 0.21 | 0.85 |
| NB | 0.89 (0.51-0.73) | 0.85 | 0.84 | 0.16 | 0.15 | 0.85 |
| Ada | 0.90 (0.59-0.78) | 0.79 | 0.93 | 0.07 | 0.21 | 0.89 |

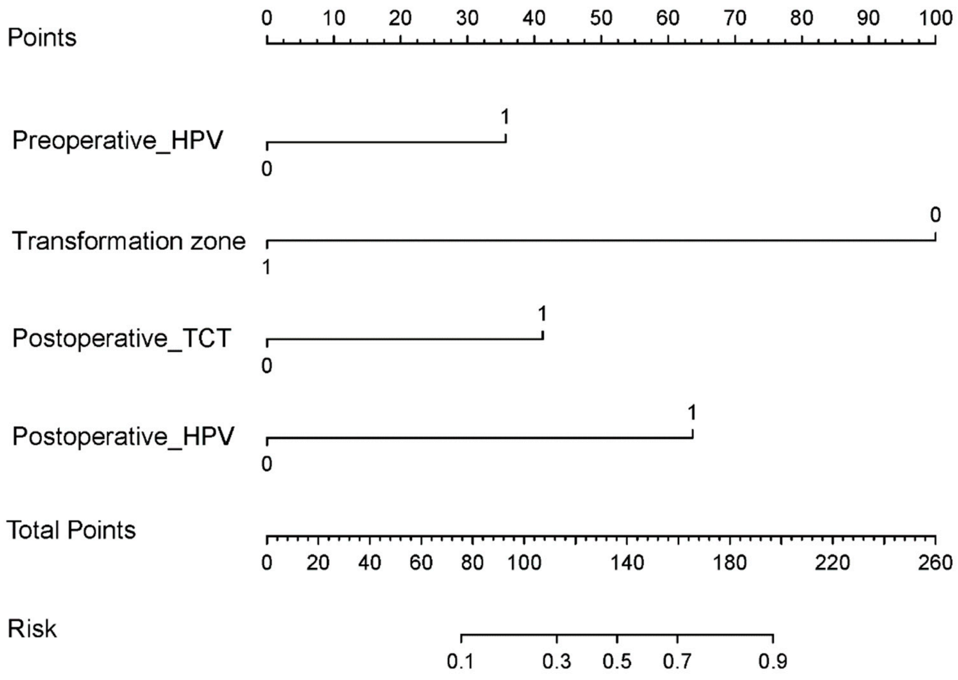


**Figure S2.** Nomogram list of the proposed model.

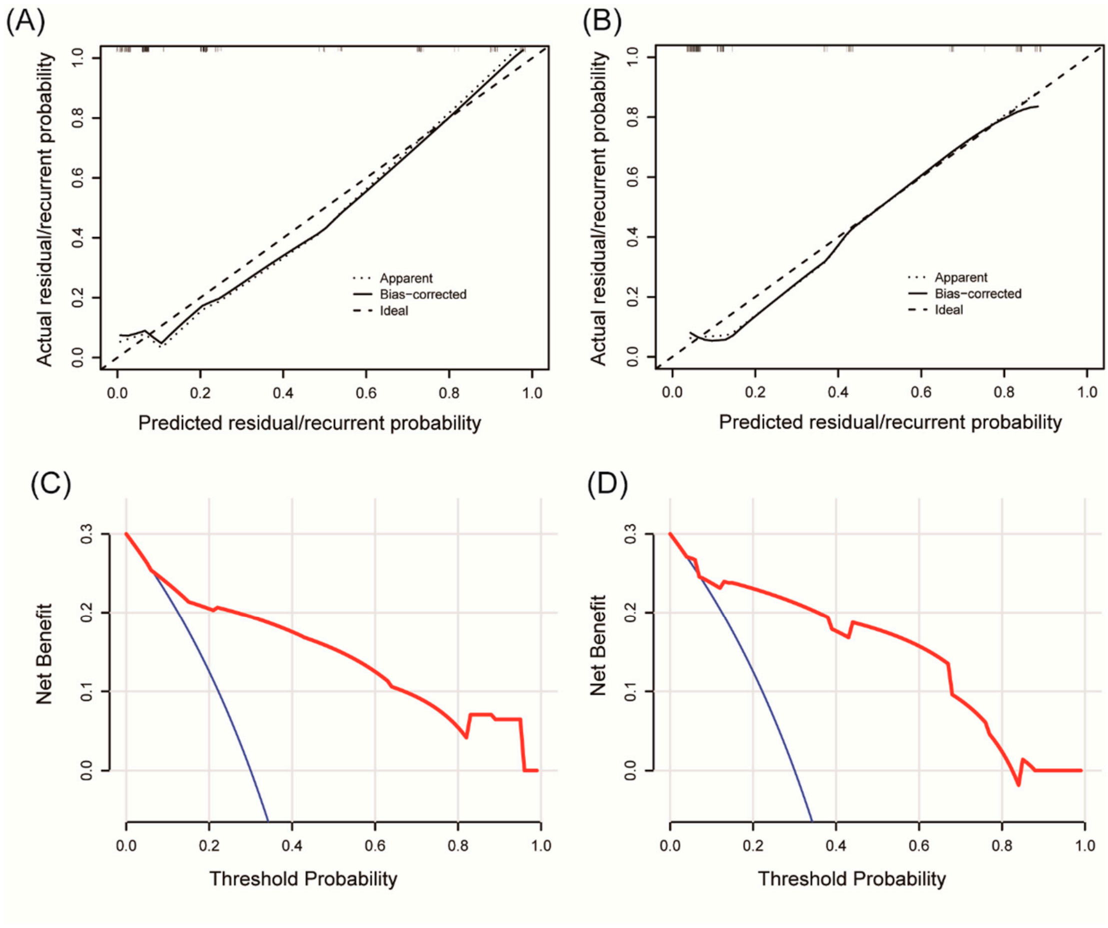


**Figure S3.** The calibration curves of the development cohort (A) and the validation cohort (B). The decision curves of the development cohort (C) and the validation cohort (D).
